# Bridging the gap: glucose transporters, Alzheimer’s, and future therapeutic prospects

**DOI:** 10.3389/fcell.2024.1344039

**Published:** 2024-01-16

**Authors:** Mai Albaik, Dalaa Sheikh Saleh, Dana Kauther, Hajira Mohammed, Shurouq Alfarra, Adel Alghamdi, Nehmat Ghaboura, Ikhlas A. Sindi

**Affiliations:** ^1^ Department of Chemistry Preparatory Year Program, Batterjee Medical College, Jeddah, Saudi Arabia; ^2^ Medicine Program, Batterjee Medical College, Jeddah, Saudi Arabia; ^3^ Department of Biology Preparatory Year Program, Batterjee Medical College, Jeddah, Saudi Arabia; ^4^ Department of Pharmacy Practice Pharmacy Program, Batterjee Medical College, Jeddah, Saudi Arabia; ^5^ Department of Biology, Faculty of Sciences, King Abdulaziz University, Jeddah, Saudi Arabia

**Keywords:** Gluts, SGLTs, facilitated diffusion, sodium-potassium ATPase, Alzheimer’s disease

## Abstract

Glucose is the major source of chemical energy for cell functions in living organisms. The aim of this mini-review is to provide a clearer and simpler picture of the fundamentals of glucose transporters as well as the relationship of these transporters to Alzheimer’s disease. This study was conducted in accordance with the Preferred Reporting Items for Systematic Reviews and Meta-Analyses (PRISMA). Electronic databases (*PubMed* and *ScienceDirect*) were used to search for relevant studies mainly published during the period 2018–2023. This mini-review covers the two main types of glucose transporters, facilitated glucose transporters (GLUTs) and sodium-glucose linked transporters (SGLTs). The main difference between these two types is that the first type works through passive transport across the glucose concentration gradient. The second type works through active co-transportation to transport glucose against its chemical gradient. Fluctuation in glucose transporters translates into a disturbance of normal functioning, such as Alzheimer’s disease, which may be caused by a significant downregulation of GLUTs most closely associated with insulin resistance in the brain. The first sign of Alzheimer’s is a lack of GLUT4 translocation. The second sign is tau hyperphosphorylation, which is caused by GLUT1 and 3 being strongly upregulated. The current study focuses on the use of glucose transporters in treating diseases because of their proven therapeutic potential. Despite this, studies remain insufficient and inconclusive due to the complex and intertwined nature of glucose transport processes. This study recommends further understanding of the mechanisms related to these vectors for promising future therapies.

## 1 Introduction

Glucose is a highly crucial energy storage molecule considered indispensable to most living cells (Bi et al., 2020). Due to its polar nature and size, it is incapable of direct diffusion into the nonpolar plasma membrane (Teymourian et al., 2020). However, to combat this and fulfill cellular energy requirements, glucose entry into cells is regulated by a family of structurally related transport proteins referred to as glucose transporters (Assimacopoulos-Jeannet et al., 1991; Holman, 2020). These glucose transporters are of two families: the facilitated diffusion glucose transporter (GLUT) and the sodium-glucose linked transporter (SGLT) (Thorens and Mueckler, 2010; Lizák et al., 2019). Solute carrier family 2 (*SLC2*) genes that express in a variety of tissues encode the proteins known as GLUTs. They are members of the major family of membrane transporters that help transport glucose across a concentration gradient (Mueckler and Thorens, 2013). In humans, 14 GLUTs have been found, which are divided into three groups (classes I, II, and III). Each group has about 500 amino acid residues and a single N-linked oligosaccharide (Assimacopoulos-Jeannet et al., 1991; Thorens and Mueckler, 2010). On the other hand, SGLTs are proteins that actively transport glucose across cell membranes (Navale and Paranjape, 2016). They use the sodium ion (Na^+^) electrochemical gradient as a driving force to transport glucose against its chemical gradient (Poulsen et al., 2015). This sodium concentration gradient is caused by the sodium-potassium ATPase as a source of chemotactic potential (Norton et al., 2017). SGLTs contain roughly 580–718 amino acids and are made up of 14 transmembrane helices with COOH and NH_2_ terminals that face the extracellular space (Navale and Paranjape, 2016).

This literature review aims to present a clearer picture of this complicated topic by studying the fundamentals of glucose transporters of both families (GLUTs and SGLTs), their structures, functions, main substrates, and locations in the human body, as well as the relationship of these transporters to the pathophysiology of Alzheimer’s disease, which is strongly correlated with high blood sugar levels. Thus, this review may help physicians, researchers, and other healthcare practitioners interested in biochemical and pharmacological research.

## 2 Methodology

The 2020 Preferred Reporting Items for Systematic Reviews and Meta-Analyses (PRISMA) guidelines were followed to conduct this literature review (Page et al., 2021).

### 2.1 Search strategy and data extraction

The search strategy was primarily for articles containing the keywords “glucose transporters” “GLUTs” and “SGLTs” and then articles in which the former terms were in combination with “Alzheimer’s disease”.

Electronic databases (*PubMed* and *ScienceDirect*) were used to search for articles containing the studied keywords, mainly published within the past 5 years (2018–2023).

### 2.2 Inclusion and exclusion criteria

**FIGURE 1:**
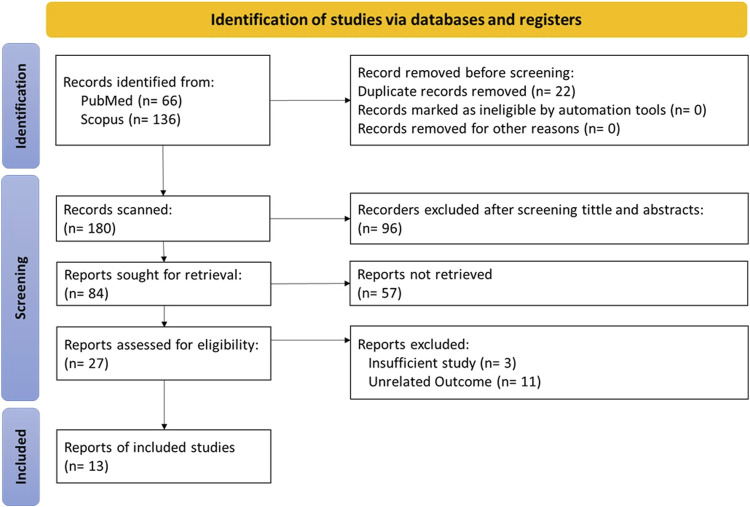
Preferred Reporting Items for Systematic Reviews and Meta-Analyses (PRISMA) flow diagram.

The inclusion and exclusion criteria for data extraction based on the PRISMA flow chart are presented in Figure 1. Our search strategy included studies related to glucose transport in both families (GLUTs and SGLTs), obtaining 66 and 133 articles from PubMed and ScienceDirect, respectively.

After applying filters, we excluded 22 duplicate studies and 96 articles by screening titles and abstracts. Excluded articles were studies published in languages other than English, studies on diseases other than Alzheimer’s disease, or studies conducted in special age groups such as children and the elderly.

After fully reviewing 84 articles, we excluded 57 articles that could not be retrieved. The result was 27 articles whose eligibility was evaluated, and we excluded 14 ineligible articles as missing or inappropriate data to finally obtain 13 articles that met our inclusion/exclusion criteria for data extraction.

## 3 Facilitated diffusion glucose transporters

**FIGURE 2:**
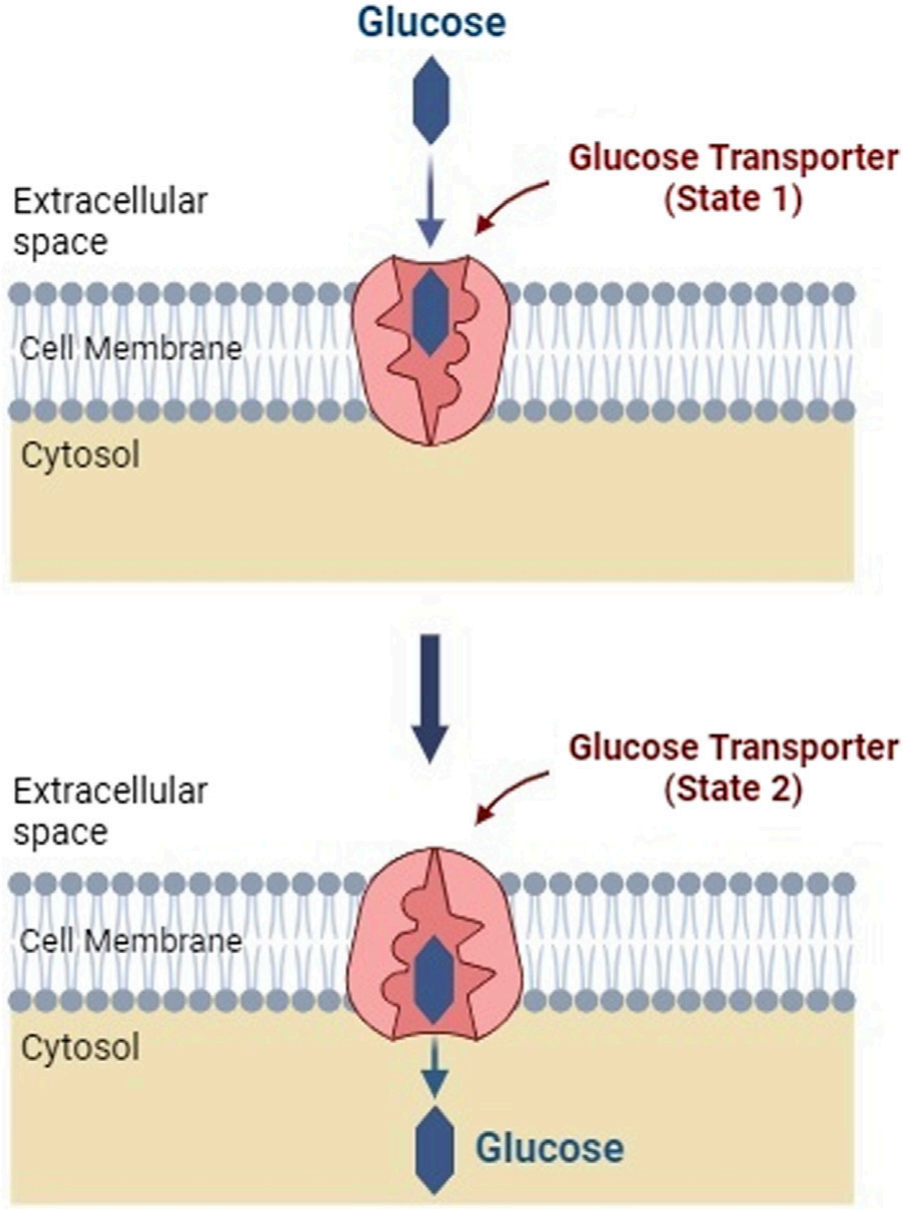
Facilitated transport of glucose into a cell membrane. Facilitated diffusion induces a conformational change in the glucose transporter protein (Created with BioRender.com).

Glucose transporters are special proteins integrated into the phospholipid bilayer of the plasma membrane by a facilitated diffusion mechanism (Pamela et al., 2005; Yan, 2015). GLUTs carry glucose molecules into the cell from an area of higher to lower concentration without using ATP energy (Navale and Paranjape, 2016). These transporters exist in the membrane in two conformational states, Figure 2. Excess cellular glucose binds to the transporter, which then changes its shape, transporting the glucose across the cell membrane (Assimacopoulos-Jeannet et al., 1991; Pamela et al., 2005; Yan, 2015).

**FIGURE 3:**
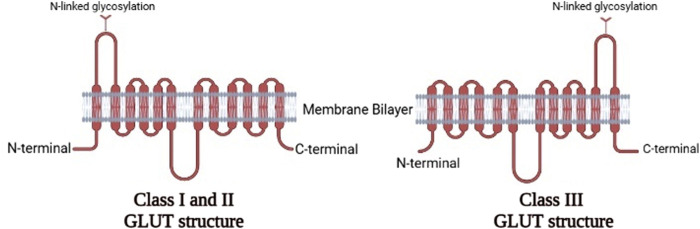
The two-dimensional representation of the structure of the three classes of GLUTs.Comparison between the structures of Class IandII GLUT proteins and Class III GLUT proteins (Created with BioRender.com).

In humans, fourteen GLUTs (GLUT-1 to GLUT-14) have been identified and classified into three classes (I, II, and III) based on sequence similarity (Assimacopoulos-Jeannet et al., 1991; Thorens and Mueckler, 2010), which are summarized in Table 1. Class I and II are structurally similar and unlike Class III due to their sites of N-linked glycosylation (McCall and Fink, 2019), as shown in Figure 3. GLUT proteins are involved in the transport of several hexoses as well as other substrates such as myo-inositol, urate, and glucosamine (Mueckler and Thorens, 2013).

**TABLE 1:** Types of GLUTs and SGLTs.

Type	Protein name	Gene name (A)	Main substrates (Km value)	Main locations	Main function	References
**GLUTs/Class I**	**GLUT1**	**SLC21**	Glucose (1–2 mM)	Many organs (brain, skeletal muscle, myocardium, erythrocytes, placenta, fetal tissues.)	Transport of glucose across the blood-brain barrier	Deng et al. (2014), Galochkina et al. (2019), Pragallapati and Manyam (2019), Veys et al. (2020), Chen et al. (2021), Drew et al. (2021), Ismail and Tanasova (2022), Vulturar et al. (2022)
	**GLUT2**	**SLC22**	Glucose (15–20 mM)	Liver, kidneys, pancreas, intestine	Involved in insulin secretion and glucose metabolism	Mueckler and Thorens (2013), Thorens, 2015; Simmons (2017), Koepsell (2020a)
			Glucosamine (0.8 mM)			
	**GLUT3**	**SLC23**	Glucose (1–2 mM)	Neurons	Regulating neuronal glucose uptake and influencing brain development and function	Simpson et al. (2008), Brown et al. (2011), Mueckler and Thorens (2013), Pragallapati and Manyam (2019), Hösli, 2020; Joshi et al. (2021), Stanirowski et al. (2021), Ismail and Tanasova (2022)
	**GLUT4**	**SLC24**	Glucose (−5 mM)	Adipocytes, muscle, heart	Responsible for insulin-regulated glucose uptake into fat and muscle cells	Mueckler and Thorens (2013), Thorens (2015), Sayem et al. (2018), Pragallapati and Manyam (2019), Chadt and Al-Hasani (2020), Ismail and Tanasova (2022)
	**GLUT14**	**SLC214**	Dehydroascorbic acid (ND^*^)	Testis	ND^*^	Amir Shaghaghi et al. (2017), Alhashim, 2022; Liu (2022)
			Glucose (ND^*^)			
**GLUTs/Class II**	**GLUT5**	**SLC25**	Fructose (5–15 mM)	Kidneys, testes, intestines	Transport, absorption and metabolism of fructose	Mueckler and Thorens (2013), Navale and Paranjape (2016), Byers et al. (2017), Tanasova and Fedie (2017), Ismail and Tanasova (2022), McComas et al. (2023)
	**GLUT7**	**SLC27**	Fructose (<0.5 mM) Glucose (<0.5 mM)	Intestine, colon, testes, prostate	Absorption of fructose and glucose	Cheeseman (2008a), Cheeseman (2008b), Navale and Paranjape (2016), Ismail and Tanasova (2022)
	**GLUT9**	**SLC29**	Fructose (=0.42 mM) Glucose (=0.61 mM) Urate (−0.6 mM)	Liver, kidney	Homeostasis of serum uric acid levels	Doblado and Moley (2009), Mueckler and Thorens (2013), Navale and Paranjape (2016), Byers et al. (2017), Lizák et al. (2019)
	**GLUT11**	**SLC211**	Fructose (0.06 mM) Glucose (−0.2 mM)	Heart, muscle, kidney, placenta, adipose, pancreas	Transportation of fructose and glucose	Szablewski (2011), Navale and Paranjape (2016), Byers et al. (2017), Gupta and Gupta (2022)
**GLUTs/Class III**	**GLUT6**	**SLC26**	Glucose (ND^*^) Fructose (ND^*^)	Brain, spleen, leukocytes	Modulator of glycolysis in inflammatory macrophages	Navale and Paranjape (2016), Maedera et al. (2019), Koepsell (2020b), Caruana and Byrne (2020)
	**GLUT8**	**SLC28**	Glucose (−2 mM)	Brain, testes, adipocytes	Facilitating the glucose transport through intracellular membranes	DeBosch et al., (2012), DeBosch et al., (2013), Chadt and Al-Hasani (2020)
	**GLUT10**	**SLC210**	Glucose (−0.3 mM)	Muscle, heart, lungs, brain, placenta, kidney, liver, pancreas	Regulation of mitochondrial function	Byers et al. (2017), Chadt and Al-Hasani (2020), Ismail and Tanasova (2022), Jian et al. (2023)
	**GLUT12**	**SLC212**	variety of hexoses	Intestine, muscles, placenta, adipose tissue	Transport of sugars	Waller et al. (2013), Byers et al., 2017; Patel (2019)
	**GLUT13 (HMIT)**	**SLC213**	Myo-inositol (−100 mM)	Neuronal tissues	Myo-inositol transporter	Navale and Paranjape (2016), Byers et al. (2017), Chadt and Al-Hasani (2020), Ismail and Tanasova (2022)
**SGLTs**	**SGLT1**	**SLC51**	Glucose (2 mM) Galactose (−2 mM)	Small intestinal cells	Absorption of glucose and galactose	Poulsen et al. (2015), Song et al. (2016), Ferrannini (2017), Koepsell (2020a), Nishimura et al. (2022)
				Proximal tubules (S2/S3) of the kidney	reabsorption of 10%–20% of filtered glucose	
	**SGLT2**	**SLC52**	Glucose (5 mM)	Proximal tubule (S1) of the kidney	reabsorption of 80%–90% of filtered glucose	Grempler et al. (2012), Harada and Inagaki (2012), Ferrannini (2017), Gyimesi et al. (2020), Wright (2021)
	**SGLT3**	**SLC54**	Glucose (20 mM)	Neurons, synapses, musculoskeletal cells, kidney, liver, brain	A glucose sensor to control glucose levels in the brain and intestines, which helps regulate bowel movements	Wright et al. (2017), Gyimesi et al. (2020)
	**SGLT4**	**SLC59**	Fructose (ND^*^) Mannose (ND^*^)	Intestine, kidney, liver, brain, lung, uterus, pancreas	Absorption and/or reabsorption of fructose, mannose, 1,5- anhydro-glucitol, and glucose	Wright et al. (2011), Navale and Paranjape (2016)
	**SGLT5**	**SLC510**	Mannose (−0.5 mM) Fructose (−0.5 mM)	Kidney cortex	Transport of mannose, fructose, glucose, and galactose	Grempler et al. (2012), Wright et al. (2017), Gonzalez-Vicente et al. (2019), Gyimesi et al. (2020)
	**SGLT6**	**SLC511**	Myo-inositol (0.12 mM) Glucose (ND^*^)	Intestine, brain, kidney	Transport of myo-inositol and glucose	Gao (2009), Baader-Pagler et al. (2018)

*ND, not determined.

### 3.1 Class I

The physiological roles of Class I GLUTs, which are GLUTs 1, 2, 3, 4, and recently included 14, have been widely studied for their impacts on metabolism and growth (Abbas et al., 2020). Each glucose transporter protein often works on specific substrates with a specific Km value (a low Km value indicates a high affinity for the substrate).

#### 3.1.1 GLUT1

The first GLUT identified was GLUT1 (encoded by the *SLC2A1* gene), which is also one of the most extensively studied membrane transport proteins (Ismail and Tanasova, 2022). The main substrate of GLUT1 is glucose with high affinity (Km of 1–2 mM) (Galochkina et al., 2019; Chen et al., 2021), and other known substrates include galactose, mannose, and glucosamine (Ismail and Tanasova, 2022). GLUT1 is ubiquitous in all tissues of the body and transports glucose across the blood-brain barrier (Pragallapati and Manyam, 2019).

Because of its characteristics, GLUT1 is mainly accountable for constitutive glucose absorption. It can be found in almost all fetal tissues, as well as many adult tissues, and is abundant in endothelial cells of blood-tissue barriers in several organs, having the highest levels of expression in proliferating cell plasma membranes throughout embryonic development (Veys et al., 2020; Vulturar et al., 2022). It is also found at high levels in the brain, skeletal muscle, and myocardium. It accounts for around 5%–10% of total membrane proteins in human erythrocytes (Pragallapati and Manyam, 2019).

As for structure, GLUT1 is a member of the major facilitator superfamily (MFS), a large family of transmembrane proteins important for the transport of small organic molecules (Drew et al., 2021). All members of the MFS share a bundle of 12 transmembrane helices, and they each have a single N-linked glycosylation site. Both the amino and carboxyl termini of the protein are exposed to the cell’s cytoplasm. GLUT1, specifically, is made up of 492 amino acids. The E329Q mutant, which stabilizes the protein’s conformation, provides the unique crystal structure of GLUT1 (Deng et al., 2014; Galochkina et al., 2019).

GLUT1 expression is influenced by levels of estrogen and progesterone in embryo implantation and is involved in maternal-placental and placental-fetal glucose transport. Additionally, GLUT1 has antioxidant activity and mitigates oxidative damage (Pragallapati and Manyam, 2019). GLUT1 has also been shown to be highly expressed in many cancer cells and is intimately relevant to the disease’s pathology.

It is worth noting that many different therapies have attempted to target GLUT1 due to its widespread prevalence, but this widespread prevalence has made it difficult to achieve specificity (Galochkina et al., 2019; Ismail and Tanasova, 2022).

#### 3.1.2 GLUT2

GLUT2 shares 55% of its amino acid sequence with GLUT1 and has a comparable shape and orientation in the plasma membrane (Ismail and Tanasova, 2022). GLUT2 was first characterized by cDNA cloning the *SLC2A2* gene from rat and human liver cDNA libraries (Mueckler and Thorens, 2013). The greatest distinguishing feature of this isoform is its unusually high Km for glucose (15–20 mM), resulting in a relatively low glucose affinity, which is required in the intestine and kidney to allow large bidirectional intracellular and extracellular fluxes (Simmons, 2017). Despite the high Km of GLUT2 for glucose uptake, the main substrate for GLUT2 is glucosamine (Km = 0.8 mM) (Koepsell, 2020a). Also, GLUT2 transports fructose, galactose and mannose (Thorens, 2015).

Highly expressed in pancreatic β-cells, as well as the basolateral membranes of the intestine, kidney epithelial cells, and hepatocytes, GLUT2 allows glucose to pass from epithelial cells into the bloodstream (Ahmad et al., 2022). In hepatocytes and β-cells, GLUT2 and glucokinase constitute a glucose-sensing system that adjusts the rate of glucose transport into the cell by controlling the secretion of glucose-stimulated insulin in pancreatic cells and regulating glucose-sensitive gene expression in hepatocytes (Simmons, 2017; Sohn and Ho, 2020). Glucose stimulates insulin secretion using an excitation-secretion coupling process: GLUT2 facilitates the entry of glucose to be phosphorylated into glucose-6-phosphate, and further oxidized to produce adenosine triphosphate (ATP). The resultant ATP closes ATP-sensitive potassium channels, thus causing a depolarization of the membrane, which prompts the opening of the voltage-gated calcium channels. Upon entering the β-cell, calcium ions trigger the exocytosis of insulin vesicles, thereby secreting insulin. On the other hand, GLUT2 in intestinal brush border cells and renal tubule cells is involved with glucose absorption and reabsorption, respectively.

While fetuses have very low levels of this isoform, GLUT2 may have significant implications for the preimplantation embryo due to its high affinity for glucosamine (Simmons, 2017). It has been found that GLUT2-dependent glucose sensing can regulate homeostatic functions such as feeding and body temperature, as well as sympathetic and parasympathetic functions in the nervous system (Thorens, 2015).

Furthermore, GLUT2 has become a target of interest for diabetes prevention and therapy due to its important involvement in carbohydrate uptake in the intestines, which inhibits glucose absorption in the intestine and lowers blood glucose levels (Thorens, 2011).

#### 3.1.3 GLUT3

In comparison to the other glucose transporters, GLUT3 has a transport capacity five times higher than that of other GLUTs. GLUT3 has a higher affinity for glucose (1–2 mM), allowing glucose transport even at low concentrations (Simpson et al., 2008; Stanirowski et al., 2021). The *SLC2A3* gene encoding GLUT3 was first cloned from a human fetal skeletal muscle cell line (Mueckler and Thorens, 2013).

GLUT3 is widely distributed in neurons, especially in pre- and post-synaptic nerve terminals and small synaptic processes (Brown et al., 2011; Ismail and Tanasova, 2022). Therefore, this transporter is considered the most important neuronal glucose transporter because it is present in both dendrites and axons, and its expression level in different parts of the brain is reflected by regional cerebral glucose consumption (Hösli, 2020).

In addition, GLUT3 has been found in varying amounts in all human tissues, with the highest concentrations found in the brain, kidneys, placenta, fetuses, sperm, and white blood cells, due to its high affinity with glucose (Simpson et al., 2008; Joshi et al., 2021; Ismail and Tanasova, 2022).

It is important to note that there are two facilitative GLUTs involved in glucose uptake in the brain: GLUT1 is mainly important for transporting glucose across the blood-brain barrier, and GLUT3 regulates glucose uptake in neurons (Pragallapati and Manyam, 2019).

#### 3.1.4 GLUT4

GLUT4 is a protein encoded by the *SLC2A4* gene in humans (Mueckler and Thorens, 2013). The main substrate of GLUT4 is glucose (Km ∼ 5 mM), and mannose, galactose, dehydroascorbic acid, and glucosamine (Ismail and Tanasova, 2022). GLUT4 is abundant mainly in striated muscle and adipose tissue, and secondarily in cardiovascular tissue (Chadt and Al-Hasani, 2020).

Contrary to other glucose transporters, GLUT4 is controlled by insulin because it is transported to the cell surface by insulin binding receptors. The responsiveness of GLUT4 to insulin signaling and membrane trafficking is due to its unique N-terminal and COOH-terminal regions (Vargas et al., 2019; van Gerwen et al., 2023). Unlike other facilitative GLUTs, GLUT4 is mostly located intracellularly in an unstimulated state and is rapidly redistributed to the plasma membrane in response to insulin and other stimuli, which can trigger a fast metabolic response and glucose uptake in a tissue (Thorens, 2015; Pragallapati and Manyam, 2019). Insulin-stimulated glucose uptake into adipose and muscle cells is GLUT4’s primary function; thus, the glucose-transport mechanism of GLUT4 can be raised to meet increased transport demands in these tissues, such as when blood glucose levels are high after ingestion of a carbohydrate-rich meal or when skeletal muscles are under greater metabolic demand during exercise (Amir Shaghaghi et al., 2017; Pragallapati and Manyam, 2019).

GLUT4 has been correlated to obesity, diabetes (type 2), and heart disease, and this association makes inhibiting this transporter a hopeful treatment option (Sayem et al., 2018; Ismail and Tanasova, 2022). GLUT4 has also been linked to Alzheimer’s disease, with several lines of research converging to suggest that the insulin-responsive glucose transporter GluT4 plays a key role in hippocampal memory processes, and that decreased activation of this transporter may underpin the cognitive impairment seen as a consequence of insulin resistance (McNay and Pearson-Leary, 2020).

GLUT4 has been the subject of more research than any other membrane transport protein because of its importance in whole-body glucose homeostasis, its complicated and elusive insulin regulation mechanism, and its disruption in various common insulin-resistant conditions (Herman et al., 2022).

#### 3.1.5 GLUT14

The most recently identified member of the GLUTs is GLUT14. GLUT14 is a transporter for dehydroascorbic acid and glucose that is expressed by the *SLC2A14* gene (Liu, 2022). GLUT14 is expressed in other organs such as the small intestine, colon, lungs, ovaries, brain, skeletal muscle, heart, kidneys, liver, blood, and placenta (Amir Shaghaghi et al., 2017). Two GLUT14 isoforms exist as a result of splice variance, and each has a unique N-terminus. Specific functions of this transporter are yet to be discovered (Alhashim, 2022).

### 3.2 Class II

Class II of facilitative glucose transporters consists of four members: GLUTs 5, 7, 9, and 11 (Carbó and Rodríguez, 2023).

#### 3.2.1 GLUT5

The first of the class II GLUT proteins to be discovered was GLUT5. The *SLC2A5* gene was initially cloned from the human small intestine (Mueckler and Thorens, 2013). Among all GLUTs, GLUT5 is a unique fructose transporter (5–15 mM) (Navale and Paranjape, 2016; McComas et al., 2023). It is located on the cell membranes of the testes, small intestines, adipose tissue, and skeletal muscle (Byers et al., 2017; Ismail and Tanasova, 2022). GLUT5 plays crucial physiological and pathological roles. Physiologically, it plays a major role in the absorption and metabolism of dietary fructose. Pathologically, it plays an important role in the pathogenesis of human gastrointestinal diseases as it is responsible for the transport of fructose, which has proven its role in a significant increase in the incidence of obesity and metabolic diseases in the whole world (Said, 2012; Song et al., 2023). Interestingly, GLUT5 may have a role in cancer treatment, as it is strongly linked to cancer development, progression, and metastasis (McQuade et al., 2013). Moreover, the increasing consumption of fructose in cancers leads to the development of GLUT5-specific inhibitors of fructose uptake (Tanasova and Fedie, 2017).

#### 3.2.2 GLUT7

GLUT7 is assigned to class II of the GLUT family based on sequence similarity. GLUT7 can transport both glucose and fructose due to its high affinity for them (Km < 0.5 mM for glucose and fructose) (Cheeseman, 2008a) and is located in cells of the small intestine, testes, colon, and prostate (Navale and Paranjape, 2016). GLUT7 has involved in developing the hypothesis that facilitated hexose transporters may have a selective filter at the exofacial opening of the translocation pore, helping to identify which hexoses can be transported, which may be useful in designing hexon analogues for use in understanding and managing cancer (Cheeseman, 2008b; Ismail and Tanasova, 2022).

#### 3.2.3 GLUT9

In humans, the GLUT9 protein is encoded by the *SLC2A9* gene (Mueckler and Thorens, 2013). It is a known transporter of fructose and can transport glucose at a low rate, as well as being a urate transporter (Byers et al., 2017). The Km for glucose was found to be 0.61 mM, the Km for fructose was 0.42 mM whereas for urate was approximately 0.6 mM (Doblado and Moley, 2009). GLUT9 has multiple isoforms in humans and is mainly found in kidney tubules, the liver, and the placenta (Navale and Paranjape, 2016; Lizák et al., 2019). In addition, it has a role in renal urate reabsorption, as inactivating mutations of GLUT9 can cause hypouricemia, and inactivation of the liver-specific GLUT9 gene can cause hyperuricemia (Rais, 2021).

#### 3.2.4 GLUT11

GLUT11 is a protein that is encoded by the *SLC2A11* gene. *SLC2A11* is a member of a family of plasma membrane proteins that mediates facilitative diffusion to transport hexoses (glucose and fructose) (Byers et al., 2017). GLUT11 is very similar to GLUT5 with 42% sequence homology, but it can transport both fructose (0.06 mM) and glucose (−0.2 mM) (Szablewski, 2011; Gupta and Gupta, 2022). GLUT11 has three isoforms in humans: GLUT11A, -B, and -C. GLUT11A is found in heart, skeletal muscle, and kidney cells; GLUT11B is found in placenta, adipose, and kidney cells; and GLUT11C is found in heart, skeletal muscle, adipose, and pancreatic cells (Navale and Paranjape, 2016). Furthermore, NCBI *RefSeq* proved the existence of a fourth isoform of GLUT11, known as GLUT11-D (Coordinators, 2016).

### 3.3 Class III

GLUTs 6, 8, 10, 12, and 13 are the five GLUTs of class III. This class, in particular, contains intracellular targeting sequences (Adekola et al., 2012).

#### 3.3.1 GLUT6

GLUT6/*SLC2A6* is a lysosomal transporter and has a low affinity for glucose and fructose (Maedera et al., 2019). It is located intracellularly, especially in the brain, spleen, and white blood cells (Navale and Paranjape, 2016). It is supposed to undergo insulin-independent endocytotic recycling (Koepsell, 2020b). GLUT6 is overexpressed in inflammatory cells and endothelial cells (Caruana and Byrne, 2020). It also modulates glycolysis in macrophages (Maedera et al., 2019).

#### 3.3.2 GLUT8

GLUT8, encoded by the *SLC2A8* gene, has a high affinity for glucose (Km ∼ 2 mM), while fructose and galactose inhibit this transport. It is mainly distributed in the brain and testes, where it has an active role in facilitating the transport of sugar through intracellular membranes (Chadt and Al-Hasani, 2020). Although the translocation of GLUT8 is hormonally regulated, it is not regulated by insulin (DeBosch et al., 2012). Furthermore, GLUT8 is overexpressed in the case of high-fat diets (DeBosch et al., 2013).

#### 3.3.3 GLUT10

GLUT10 is encoded by the *SLC2A10* gene (Byers et al., 2017). GLUT10 has a 35% similarity to GLUT2, and it can also transport glucose (−0.3 mM) (Ismail and Tanasova, 2022; Jian et al., 2023). GLUT10 is located in skeletal muscle tissue and the heart, lungs, brain, placenta, kidney, liver, and pancreas (Chadt and Al-Hasani, 2020; Jian et al., 2023). GLUT10 knockout has been previously associated with impaired mitochondrial function, but the underlying mechanism for this association remains unknown (Ismail and Tanasova, 2022).

#### 3.3.4 GLUT12

*SLC2A12* encodes a GLUT12 transporter that facilitates the transport of a variety of hexoses (Byers et al., 2017). GLUT12 is expressed in the small intestine, skeletal muscles, placenta, and adipose tissue (Patel, 2019). GLUT12 is similar to GLUT10 in sequence and GLUT4 in action. The expression of GLUT12 on the surface of cardiomyocytes is not insulin-dependent, suggesting that it is a basal glucose transporter (Waller et al., 2013). GLUT12 offers a promising new therapeutic pathway due to its importance and role in breast cancer (Ismail and Tanasova, 2022) and Alzheimer’s disease (Gil-Iturbe et al., 2020).

#### 3.3.5 GLUT13

GLUT13 is encoded by the *SLC2A13* gene (Ismail and Tanasova, 2022). GLUT13 is defined as a proton (H^+^) myo-inositol cotransporter (HMIT) that has the specificity of transporting myo-inositol, inositol triphosphate, and related stereoisomers (Byers et al., 2017). GLUT13 differs from the other 13 transporters in that it does not transport glucose or fructose, but its only substrate is myo-inositol (Km ∼ 100 mM), and it depends on the pH of the medium (Ismail and Tanasova, 2022). GLUT13 is mainly localized in neuronal tissues; its action is controlled by changes in cell membrane potential (Navale and Paranjape, 2016; Chadt and Al-Hasani, 2020).

## 4 Sodium-glucose linked transporters

Sodium-glucose linked transporters (SGLTs) belong to the *SLC5* family of active glucose transporters (Ferrannini, 2017). This is an energy-requiring process that transports glucose “against” a concentration gradient, that is, from low glucose concentrations outside the cell to higher concentrations within the cell (Poulsen et al., 2015). SGLTs can transport glucose by utilizing energy generated from the sodium-potassium pump (Navale and Paranjape, 2016; Ferrannini, 2017). Glucose transport via SGLTs is therefore active, contrasting the passive action of GLUTs, and is coupled with sodium uptake (Carbó and Rodríguez, 2023).

SGLTs exist in six isoforms in humans (SGLT-1 to SGLT-6) (Sano et al., 2020), Table 1.

### 4.1 SGLT1

SGLT1 is a human protein that is encoded by the *SLC5A1* gene (Poulsen et al., 2015). It was found that mutations in the *SLC5A1* lead to glucose-galactose malabsorption (Ferrannini, 2017; Sano et al., 2020). SGLT1 is a high affinity, low capacity transporter. The main substrates of SGLT1 are glucose and galactose (Km of 2 mM) (Song et al., 2016). SGLT1 facilitates the uptake of dietary glucose and galactose, and works to prolong glucose excursion to maximize intestinal sugar uptake (Nishimura et al., 2022).

SGLT1 is also mainly located in the small intestine. It is also found in the distal part of the proximal tubule (S3) in the kidney and is responsible for the reabsorption of 10%–20% of filtered glucose (Ferrannini, 2017; Koepsell, 2020a).

### 4.2 SGLT2

The preferred substrate for SGLT2 is glucose. SGLT2 is a low affinity, high capacity glucose co-transport protein (KM of 5 mM) (Wright, 2021). SGLT2 is located in the early proximal tubules (S1 and S2) of the kidney and is responsible for the reabsorption of 80%–90% of filtered glucose (Harada and Inagaki, 2012; Ferrannini, 2017). The gene encoding SGLT2 is *SLC5A2* and a mutation in this gene leads to glucosuria (Ferrannini, 2017; Sano et al., 2020).

**FIGURE 4:**
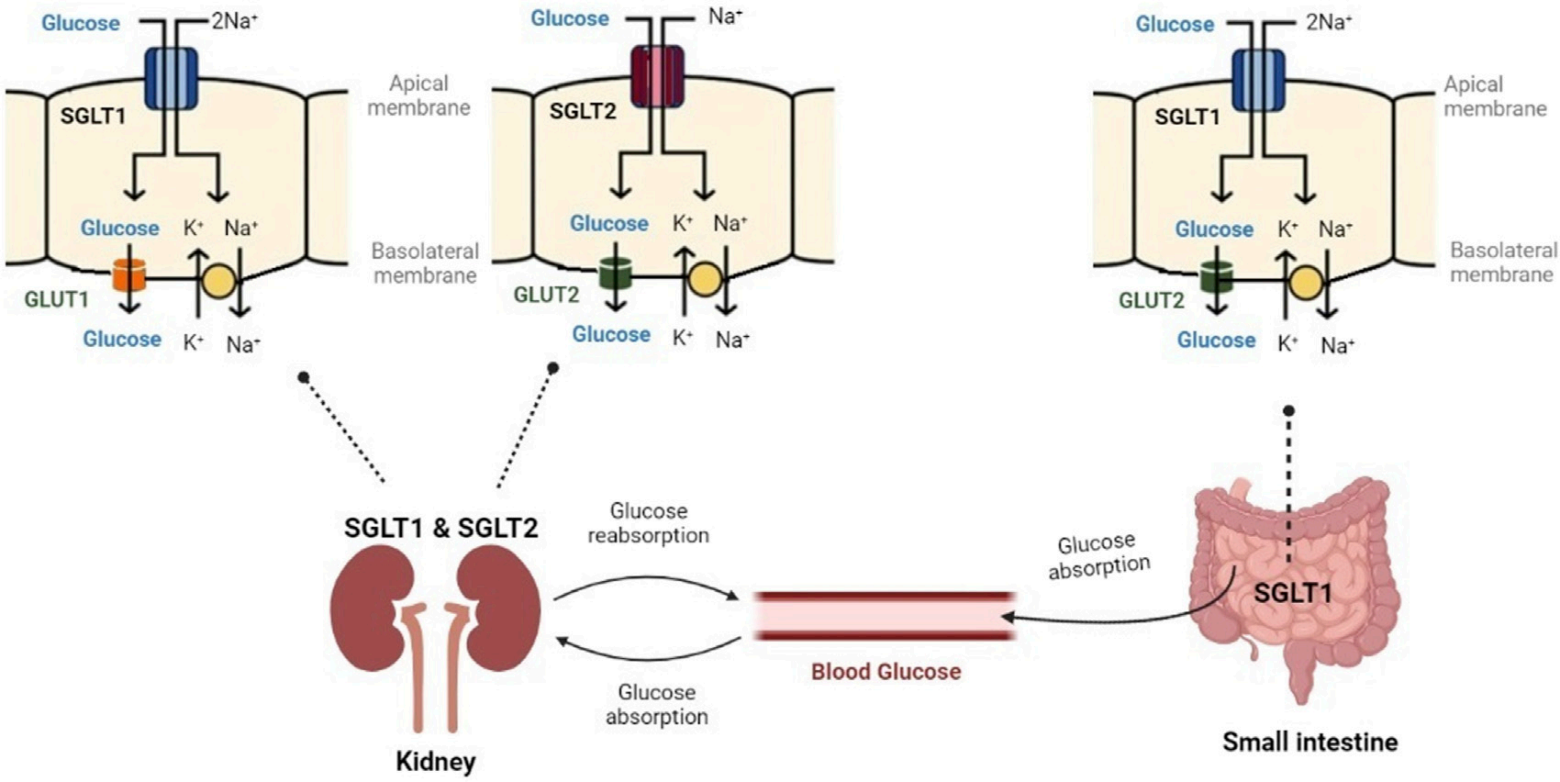
Glucose controlling through SGLT1 and SGLT2. SGLT1 has a high affinity for glucose and transports sodium and glucose, mainly in the small intestine. SGLT2 has a low affinity for glucose and transports sodium and glucose in the kidney (Created with BioRender.com).

SGLT2 and SGLT1 are of high physiological importance. Figure 4 displays their role in glucose control. In the small intestine, SGLT1 transports sodium and glucose using a 2:1 stoichiometry. In the kidney, SGLT2 transports sodium and glucose using a 1:1 stoichiometry (Sano et al., 2020). SGLT2 and SGLT1 have been extensively researched for their pathophysiological roles in diabetes mellitus and end-stage renal disease (Wright et al., 2011; Nishimura et al., 2022).

### 4.3 SGLT3

SGLT3, *SLC5A4*, binds glucose with low infinity (Km = 20 mM) (Gyimesi et al., 2020). SGLT3 is located in enteric neurons, synapses, musculoskeletal cells, and the brain. It plays the role of a glucose sensor to control glucose levels in the brain and intestines, which helps regulate intestinal motility (Wright et al., 2017).

### 4.4 SGLT4

SGLT4/*SLC5A9* is a crucial transporter for mannose, fructose, glucose, and 1,5-anhydroglucitol (Tazawa et al., 2005). It was found in the intestine, kidney, liver, brain, lung, uterus, and pancreas (Navale and Paranjape, 2016).

### 4.5 SGLT5

SGLT5 is a protein encoded by the *SLC5A10* gene in humans. It is transporter of mannose and fructose (Km = 0.45 and 0.62 mM, respectively). SGLT5 demonstrates the usual functional traits of the *SLC5* glucose transporter family members, including the inhibition of the transport mechanism by phlorizin and Na^+^-dependence of the transport process (Grempler et al., 2012; Gyimesi et al., 2020). SGLT5 has additional physiological functions that have not yet been discovered. Although not found in any other tissues, SGLT5 is strongly expressed in the kidney (Wright et al., 2017; Gonzalez-Vicente et al., 2019).

### 4.6 SGLT6

*SLC5A11* is a protein-coding gene for SGLT6 which is an active transporter of myo-inositol (Km = 0.12 mM) and glucose (Gao, 2009; Baader-Pagler et al., 2018) in the brain, kidney, and intestine (Baader-Pagler et al., 2018).

As a mean of countering the pathological activity of these transporters, SGLT-inhibiting drugs have been developed. SGLT2 inhibitors have demonstrated great success in the treatment of type 2 diabetes mellitus, in addition to having been found to have advantageous effects on the cardiovascular system, kidneys, and Alzheimer’s disease (Rieg and Vallon, 2018; Esterline et al., 2020; Nishimura et al., 2022).

## 5 Alzheimer’s disease associated with glucose transporters

Alzheimer’s disease (AD) is defined as a progressive neurodegenerative disorder described by varying levels of cognitive impairment based on memory loss, confusion, and problems with sensory processing (Scheltens et al., 2021).

The earliest stages of AD begin to manifest several years—typically, decades or more—before the identification of any serious symptoms, studies detailing the histopathological features of the birth and progression of AD illustrate neurotoxic changes (Pahlavani, 2023). The neurotoxic changes are generated by prolonged exposure to damage signals that produce cytotoxic factors and pro-inflammatory cytokines that cause the phenomenon of neuroinflammation (Morales et al., 2014). The hypothesis is that inflammation in the brain increases the risk of AD by producing amyloid beta peptide (Aβ), observed in the presence of senile extracellular amyloid plaques (APs), and the formation of neurofibrillary tangles (NFTs) due to the buildup of hyperphosphorylated tau protein. The accumulation of these neurotoxic substances causes neural death and, eventually, the shrinking of the brain (Lopez et al., 2019).

Another distinctive feature of AD is synapse loss, as synapse formation is an important process in the nervous system throughout a healthy person’s life. Synapses facilitate neuronal connectivity and enable memory formation and retrieval (Szablewski, 2021a). We constantly lose and then regenerate these crucial brain connections throughout our lifetimes. A healthy brain maintains a net number of synapses because the number of new synapses equals the loss of old ones. Learning and memory are based on a process known as synaptogenesis, which is the formation of new synapses (Zhu et al., 2020). Loss of synapses may be explained by dysregulated insulin/IGF-1 signaling, as brain insulin resistance or dysregulation contributes to neurodegeneration in AD (McEwen and Reagan, 2004; Szablewski, 2021a).

Despite the fact that the precise mechanism of AD pathogenesis is still unknown, damaged mitochondria probably play important roles in the pathogenesis of AD (Bhatia et al., 2022; Wang et al., 2020; Dewanjee et al., 2022/11). The primary evidence of mitochondrial malfunctioning comes from the abnormal energy hypometabolism observed in certain parts of the brain of AD patients (Bhatia et al., 2022). It is believed that a healthy pool of mitochondria not only supports neuronal activity by providing adequate energy supply and other related mitochondrial functions to neurons but also guards neurons by reducing mitochondria-related oxidative damage (Wang et al., 2020).

Another attempt to explain the mechanism examines the relationship between AD and changes in insulin and GLUT functions (Koepsell, 2020c). Huge number of peer review publications express that midlife obesity is a risk factor for the development of diabetes and insulin resistance which can lead to the hyperpolarization of the tau proteins and the similar effects are seen in central insulin resistance resulting in the deletion of neuronal insulin receptors. Further the mechanism may involve the activation of GSK3β which are evident that defective insulin signaling may invoke tau hyperpolarization and dysfunction (Blum and Buée, 2013). It is clearly evident that the brain derives energy through the neurons by the utilization of glucose in the presence of insulin. Specifically, hypothalamus may be considered as the powerhouse of the brain in generating the energy needed for the proper functioning additionally insulin sensitive and responding centers are present in the forebrain responsible for memory and as per the recent studies it was established that the insulin has neuroprotective and memory enhancing activity. Henceforth, it can be predicted that dysregulation of insulin may impair the memory related logistics in the brain eventually leading to memory loss as in AD (De Felice et al., 2014).

In addition to this explanation the inulin sensitive receptors present on the surface of the blood brain barriers (BBB) may help in the signal transduction throughout the brain. Insulin from the peripheral tissues enters the brain also through the endocytosis-exocytosis pathway. Sensitization to these receptors and mechanisms may impair the transportation of insulin across the brain leading to the memory impairment issues as seen in AD. Downstreaming pathways in the brain like PI3K routes may produce the synaptic plasticity and proves the link between the insulin sensitivity and AD (Diehl et al., 2017).

### 5.1 Overview of physiology of normal brain glucose transporters

The brain’s primary energy source is glucose, but it also obtains energy from consuming ketone bodies and minimal other sugars such as fructose (Beltrán et al., 2012). Not only that, but glucose is also needed for the hexosamine biosynthesis pathway (HBP), which is needed for OGlcNAcylation, which is an important process for brain health. The brain cannot make or store glucose, so it needs a constant supply of it coming in through glucose transporters in the blood-brain barrier (BBB) (Beltrán et al., 2012).

Ten of the fourteen known GLUTs have been found in the nervous system. Four of these (GLUTs 1, 2, 3, and 4) are most directly affected by and/or have a function involved in the pathology that has been talked about (Shah et al., 2012; Szablewski, 2017).

GLUT1 and 3 are most predominant in their quantity and functional contribution to the brain. GLUT1 is expressed in endothelial cells of the BBB, where its levels are highly determinant of glucose uptake levels, and notably unexpressed in neurons. Contrastingly, GLUT3 is generally neuron-specific: glucose uptake is increased by the fusion of GLUT3 with the neuron plasma membrane. (Chen et al., 2022). More poorly studied is GLUT2, which is found in astrocytes and tanycytes and is thought to be involved in cerebral glucose sensing (López-Gambero et al., 2019). Lastly, GLUT4 is an insulin-sensitive GLUT in the brain, meaning that its expression and translocation are upregulated by insulin (Milstein and Ferris, 2021). Though present in major brain zones such as the hypothalamus, cerebellum, and hippocampus, the exact function of GLUT4 in the brain remains unknown. Hypotheses propose that GLUT4 may provide further glucose to neurons in states of high-energy demand, regulate glucose uptake in certain brain regions, or be involved in glucose sensing (Szablewski, 2017; Szablewski, 2021a).

### 5.2 Observations on glucose transporters in Alzheimer’s

Although the literature on the specifics of what causes AD remains poor, research provides a promising, albeit incomplete, groundwork for an association between glucose activity and the formation of APs and NFTs.

Perhaps the most significant driving factor in the disruption of the brain’s chemistry in AD is the disruption of the levels of insulin, which may be a cause or effect of neurodegeneration but is nonetheless a significant participant in its progressive nature (Sharma et al., 2022). In the brain, insulin activates the phosphoinositide-3 kinase (PI3-K) and mitogen-activated protein kinase (MAPK) pathways that, in turn, activate protein kinase B (PKB), a protein that involves a fundamental role in the translocation of GLUT4 (Szablewski, 2021a). Consequent to the insulin resistance that accompanies AD, impairment in GLUT4 translocation decreases the uptake of glucose, disrupting normal glucose metabolism and, thus, inducing an increase in oxidative and endoplasmic stress and the generation of reactive species that damage DNA, RNA, and proteins (Szablewski, 2017). This stress and imbalance, along with the absence of insulin that would otherwise act to regulate the degradation and processing of the amyloid-β protein precursor (AβPP), consequently lead to an increase in the expression of the AβPP gene, resulting in an accumulation of amino acids that form larger insoluble fibers upon aggregation, called AβPP-Aβ oligomeric fibrils. As described in AD pathology, further aggregation of AβPP-Aβ oligomeric fibrils over time forms the neurotoxic APs that factor into the stunting of neural development, learning processes, and memory (Matioli and Nitrini, 2015). Notably, most studies are inconclusive as to which precedes: insulin resistance or AβPP-Aβ aggregation. This is because AβPP-Aβ competes with insulin and makes insulin receptors (IRs) less sensitive, which lowers insulin affinity. At the same time, insulin resistance causes AβPP-Aβ to build up (Shah et al., 2012). A further demonstration of insulin’s effect on AD is its role in tau phosphorylation. Under normal conditions, tau contributes to the assembly and stabilization of neuron microtubules; however, its hyperphosphorylation, which causes the tau proteins to tie themselves into NFTs, is primarily responsible for the symptoms of AD. This occurs as a decline in insulin inhibits the activity of the PI3-K pathway that regulates hypoxia-inducible factor 1 (HIF-1), a protein complex that activates the transcription of GLUTs 1 and 3. Decreased levels of HIF-1, in turn, cause a mass downregulation of GLUTs 1 and 3; the resulting hypometabolism of glucose downregulates OGlcNAcylation, which varies inversely as tau phosphorylation (Szablewski, 2017; Kyrtata et al., 2021).

Furthermore, a large upregulation of—and in one study, twice as many—GLUT2 was observed in AD brains, which scientists hypothesize is a mechanism of compensation for the drastic decrease in cerebral glucose supply (Szablewski, 2017).

One of the glucose transporters that has proven a role in Alzheimer’s disease is GLUT12, as a study by Pujol-Gimenez et al., in 2014 demonstrated that the expression level of GLUT12 is much higher in Alzheimer’s patients (Pujol-Gimenez et al., 2014).

### 5.3 Glucose transporters and outlook on treatment for Alzheimer’s

In light of AD’s etiology, new treatment approaches ought to be investigated. Certain hypoglycemic medications that treat problems with glucose metabolism may encourage brain regeneration and metabolism, greatly enhancing memory and cognitive function (Patrone et al., 2014; Wang et al., 2023).

Glucose transporters may play a role as potential targets for the treatment of Alzheimer’s disease. GLUT1 and GLUT3, two glucose transporters in neurons, transport the glucose down its gradient (Deng and Yan, 2016). Decreased expression of GLUT1 and GLUT3 is linked to decreased glucose absorption. As a result, it's hypothesized that improving glucose transport into neurons could be an effective treatment for AD (Szablewski, 2021b).

By controlling insulin pathways and energy metabolism, hypoglycemic medications such as antidiabetic drugs (e.g., metformin), sodium-glucose cotransporter 2 (SGLT2) inhibitors, sodium-glucose cotransporter 1 (SGLT1) agonists, Dipeptidyl peptidase IV (DPP4) inhibitors, Glucagon-like peptide-1 receptor (GLP-1 RAs) agonists, liposomes, insulin, and Small interfering RNA (siRNA) have been shown to be effective in treating Alzheimer’s disease (Szablewski, 2017; Szablewski, 2021a; Wang et al., 2023).

Among these medications mentioned above, this mini-review will focus on SGLT2 inhibitors that work by increasing urinary glucose excretion. SGLT2 inhibitors block the action of SGLT2 proteins and decrease glucose reabsorption. They control the blood glucose levels by decreasing the renal glucose threshold. One of the advantages of this type of inhibitor is that it is independent of insulin (Rieg and Vallon, 2018; Wright, 2021). The American Diabetes Association (ADA) approved four single SGLT2 inhibitors: Canagliflozin (Invokana), Dapagliflozin (Farxiga), Empagliflozin (Jardiance), and Ertugliflozin (Steglatro) (Simes and MacGregor, 2019).

Finally, it is worth mentioning that regular exercise is very important to achieve the therapeutic effect as well (Pahlavani, 2023). Nevertheless, additional studies are necessary to completely comprehend the treatment goals.

## 6 Conclusion

As has been previously emphasized, glucose transporters are involved in crucial physiological roles and pathophysiological mechanisms since the maintenance of bodily glucose levels is critical. The different locations, functions, and chemical make-up of GLUTs and SGLTs help keep energy-using processes going by controlling glucose transport as blood glucose levels change. Any defect in these transporters may translate into a disruption of normal functioning, such as Alzheimer’s disease, which may be caused by a significant downregulation of GLUTs most closely linked to insulin resistance in the brain.

The strength of this review is that it clearly provides a comprehensive classification of the different types of glucose transporters, and it also provides a better and deeper understanding of glucose transporters and their relationship to Alzheimer’s disease. This review may assist researchers and healthcare practitioners in their work and answer their key questions about relevant research. The limitations of this review are that it needs to include more detail and discussion about the molecular mechanisms, relationships with neurological diseases, and potential future directions.

Overall, research on a number of GLUT and SGLT mechanisms and how they might be involved in diseases is still poor and not very clear. This has slowed down the progress toward a better understanding of the role of glucose transporters and, in turn, research that uses them now. So, focusing on the more basic parts needed to understand GLUTs, SGLTs, and how they work with other parts of the body that keep glucose levels stable is important for unlocking the huge potential that glucose transporter research has for creating ways to detect and treat diseases that are caused by high glucose levels.
